# Correlation of intraoperative blood pressure variability and postoperative delirium in elderly hip fracture surgery

**DOI:** 10.1038/s41598-025-00019-0

**Published:** 2025-04-29

**Authors:** Peng Yang, Yu Fan, Wanyun Tang

**Affiliations:** 1 Department of Orthopedics, Zigong First People’s Hospital, No. 42, Yizhi Road, Shangyihao Street, Zigong, 643000 Sichuan People’s Republic of China; 2 Department of Anesthesiology, Zigong First People’s Hospital, 643000 Zigong, People’s Republic of China

**Keywords:** Hip fracture, Blood pressure variability, Postoperative delirium, Risk factor, Geriatric, Medical research, Risk factors, Signs and symptoms, Trauma

## Abstract

This study investigates the relationship between intraoperative blood pressure variability (BPV) and postoperative delirium (POD) after hip fracture surgery in geriatric patients. A retrospective analysis was conducted on 1002 geriatric patients who underwent hip fracture surgery. Intraoperative BPV was mainly quantified using the coefficient of variation in mean arterial pressure (CV-MAP). *Patients were stratified into two groups (CV-MAP* ≤ *10% vs.* > *10%). Propensity score matching (PSM) balanced baseline characteristics*. Multivariable logistic regression evaluated the association between CV-MAP and POD. Restricted cubic spline (RCS) analysis examined dose–response relationships. Subgroup analyses and interaction tests were conducted to examine effect modifications. POD occurred in 198 patients (19.8%). Patients with CV-MAP > 10% showed a significantly higher occurrence of POD than those with CV-MAP ≤ 10%, both before (24.6% vs. 16.4%, p < 0.001) and after PSM (25.2% vs. 18.9%, p = 0.032). Adjusted logistic regression confirmed CV-MAP > 10% as an independent predictor of POD (adjusted OR: 1.45, 95% CI 1.03–2.03, p = 0.033). RCS analysis revealed a nonlinear positive association between CV-MAP and POD risk. Subgroup analyses identified significant interactions between CV-MAP and variables such as age and ASA classification (p < 0.05). Elevated intraoperative BPV is independently associated with an increased risk of POD in elderly hip fracture patients, with nonlinear effects and potential modifiers. These findings underscore the importance of individualized blood pressure management to mitigate POD risk.

## Introduction

Hip fractures are a major health concern among geriatric patients, contributing to significant morbidity, mortality, and healthcare costs globally^1^. Each year, an estimated 1.6 million hip fractures occur worldwide, a number projected to increase to 4.5 million by 2050 due to the aging population^2^. In the United States alone, hip fractures account for over 300,000 hospital admissions annually, with associated healthcare expenditures exceeding $20 billion^3,4^. Hip fractures often necessitate surgical intervention to restore mobility, reduce pain, and prevent complications such as pressure ulcers and thromboembolism^5^.

However, elderly patients undergoing hip fracture surgery frequently present with multiple comorbidities, such as hypertension, diabetes, and cardiovascular disease, as well as frailty and diminished physiological reserves^6,7^. These factors increase their vulnerability to perioperative complications, including POD, which affects 10–60% of patients in this population^5,8–10^.

POD is a common and serious neurocognitive disorder characterized by acute and fluctuating disturbances in attention, awareness, and cognition^11,12^. Its incidence after hip fracture surgery ranges from 28 to 61%, depending on patient characteristics and perioperative factors^13–15^. POD is associated with prolonged hospital stays, functional decline, increased healthcare costs, and a two- to threefold rise in one-year mortality rates^16–19^. Despite its clinical significance, the pathophysiology of POD is not yet fully elucidated, and effective preventive strategies are limited. Identifying modifiable risk factors, particularly those related to perioperative management, is a priority in reducing POD incidence.

Intraoperative BPV has emerged as a potential modifiable risk factor influencing POD risk^20–23^. BPV refers to fluctuations in blood pressure during surgery, which can disrupt cerebral autoregulation, leading to hypoperfusion or hyperperfusion of the brain^24^. These disturbances may contribute to neuroinflammation, oxidative stress, and endothelial dysfunction, which are implicated in the pathogenesis of POD^25^. Previous studies have demonstrated an association between BPV and adverse perioperative outcomes, including organ dysfunction, cognitive impairment, and mortality^26–28^. nHowever, evidence linking BPV specifically to POD remains constrained by small sample sizes and heterogeneous study designs, with most studies focusing on absolute blood pressure values rather than dynamic changes. Furthermore, the underlying mechanisms by which BPV contributes to POD and the potential modifying effects of patient characteristics and comorbidities warrant further investigation.

This study aims to clarify the association between intraoperative BPV and POD in geriatric patients undergoing hip fracture surgery by utilizing a propensity score-matched cohort.

## Methods

### Data sources and patient

This retrospective cohort study was conducted using electronic medical records from a tertiary hospital between January 2021 and January 2025. The study included geriatric patients (≥ 65 years) who underwent hip fracture surgery. The Institutional Review Board (IRB) of the tertiary trauma center approved this study, which used anonymized clinical data. Inclusion criteria were: (1) age ≥ 65 years; (2) X-ray or CT diagnosis; (3) and surgical confirmation. Exclusion criteria: (1) No surgical intervention; (2) Age < 65 years; (3) Pathological, old, multiple or open fractures ;(4) Severe infections or severe cardiac, hepatic or renal dysfunction; (5) Local anesthesia or neuraxial anesthesia; (6) Incomplete data.

### Data collection

The data for this study were sourced from our hospital health information system. A wide range of indicators was collected, encompassing demographic characteristics, comorbidities, operation-related factors, and laboratory findings. Demographic data included gender, age, smoking status, and alcohol consumption. Comorbidities assessed were hypertension, diabetes, chronic obstructive pulmonary disease (COPD), cardiovascular disease, cerebral infarction, dementia, intracerebral hemorrhage, chronic liver disease, chronic kidney disease, tumors, and chronic steroid use. Operation-related factors included the type of fracture (femoral neck fracture, intertrochanteric fracture, or subtrochanteric fracture) and the type of surgery (total hip arthroplasty, hemiarthroplasty, intramedullary nail fixation, internal fixation with a steel plate, or internal fixation with hollow nails). Other surgical variables considered were intraoperative blood loss, intraoperative time, intraoperative blood pressure, transfusion status, postoperative ICU admission, bedridden time, and the American Society of Anesthesiologists (ASA) classification (I–II or III–IV). Laboratory findings included white blood cell (WBC) count, neutrophil (NEU) count, hemoglobin (HGB) level, potassium (K), sodium (Na), blood glucose, creatinine (Cr), albumin, and D-dimer levels. These indicators provided a comprehensive dataset for the analysis.

For patients with multiple laboratory measurements prior to surgery, the values recorded closest to the time of surgery were selected for analysis. Before initiating the study, three researchers underwent specialized training to ensure consistency and accuracy in data collection. Two researchers independently gathered data, including information on POD events. Discrepancies were resolved through collaborative discussion, with final decisions made by the senior researcher when required.

### Exposure

The primary exposure was intraoperative blood pressure, and we collected intraoperative systolic blood pressure and diastolic blood pressure at 10-min intervals and calculated the MAP. Systolic blood pressure below 40 mmHg or above 300 mmHg and diastolic blood pressure below 20 mmHg or above 150 mmHg were set to missing and excluded from analysis^29^. BPV was quantified by calculating the standard deviation (SD) and a coefficient of variation (CV) of blood pressure. These indicators include Mean Systolic Blood Pressure (Mean-SBP), Standard Deviation of Systolic Blood Pressure (SD-SBP), Coefficient of Variation of Systolic Blood Pressure (CV-SBP), Mean Diastolic Blood Pressure (Mean-DBP), Standard Deviation of Diastolic Blood Presssure (SD-DBP), Coefficient of Variation of Diastolic Blood Pressure (CV-DBP), Mean Arterial Pressure (Mean-MAP), Standard Deviation of Mean Arterial Pressure (SD-MAP), and Coefficient of Variation of Mean Arterial Pressure (CV-MAP). The formulas of those variables are below: *Blood pressure variability (BPV) was quantified using the standard deviation (SD) and coefficient of variation (CV) of intraoperative mean arterial pressure (MAP), consistent with recommendations from the European Society of Hypertension, which advocates SD and CV as optimal metrics for assessing short-term BPV due to their complementary strengths*^30^*. SD captures absolute fluctuations, while CV normalizes variability relative to mean pressure (CV* = *[SD/mean]* × *100%), mitigating confounding by baseline blood pressure levels—a critical consideration in elderly cohorts with heterogeneous hemodynamic profiles*^31^*.*

$$\text{Mean}{}_{\text{SBP}}=\frac{\sum {\text{SBP}}_{i}}{\text{N}},$$

$$\text{SD}\_\text{SBP}=\sqrt{\frac{\sum {\left({\text{SBP}}_{i} -\text{Mean}\_\text{SBP}\right)}^{2}}{N} },$$

$$\text{CV}\_\text{SBP}=\frac{\text{SD}\_\text{SBP}}{\text{Mean}\_\text{SBP}} \times 100\text{\%}.$$

Where SBP_i_ represents individual systolic blood pressure readings, and N is the total number of readings.

$$\text{Mean}\_\text{DBP}=\frac{\sum {\text{DBP}}_{i}}{\text{N}},$$

$$\text{SD}\_\text{DBP}=\sqrt{\frac{\sum {\left({\text{DBP}}_{i} -\text{Mean}\_\text{DBP}\right)}^{2}}{N} },$$

$$\text{CV}\_\text{DBP}=\frac{\text{SD}\_\text{DBP}}{\text{Mean}\_\text{DBP}} \times 100\text{\%}.$$

Where DBP_i_ represents individual diastolic blood pressure readings, and N is the total number of readings.

$$\text{Mean}\_\text{MBP}=\frac{\sum {\text{MAP}}_{i}}{\text{N}},$$

$$\text{SD}\_\text{MAP}=\sqrt{\frac{\sum {\left({\text{MAP}}_{i} -\text{Mean}\_\text{MAP}\right)}^{2}}{N} },$$

$$\text{CV}\_\text{MAP}=\frac{\text{SD}\_\text{MAP}}{\text{Mean}\_\text{MAP}} \times 100\text{\%}.$$

Where MAPi represents individual MAP readings, and N is the total number of readings.

In this study, intraoperative BPV was mainly quantified using the CV_MAP. It normalizes the standard deviation of MAP to its mean, providing a percentage that quantifies the relative fluctuation in blood pressure^32^. Patients were stratified into two groups based on a CV-MAP threshold (CV-MAP ≤ 10% vs. CV-MAP > 10%) determined from previous studies and clinical significance^33^. Additionally, we divided the CV-MAP into three groups (T1 group (CV-MAP ≤ 7.56%), T2 group (CV-MAP = 7.56%-10.81%), T3 group (CV-MAP > 10.81%)) based on tertiles to better reveal the impact of intraoperative BPV on POD.

### Outcome

The primary outcome of this study is delirium within seven days after surgery. The diagnostic criteria for POD are primarily based on the Diagnostic and Statistical Manual of Mental Disorders (DSM-5) by the American Psychiatric Association^34^. Key features include a disturbance in consciousness with reduced environmental awareness, attention deficits, and acute changes in cognitive function such as memory impairment, disorientation, or perceptual disturbances. These symptoms typically develop rapidly (within hours to days), fluctuate in severity, and are often associated with underlying physiological factors, such as postoperative changes. Importantly, other potential causes, including dementia or psychiatric disorders, must be excluded to confirm the diagnosis. Two board-certified neurologists independently reviewed medical records to diagnose POD using DSM-5 criteria. Discrepancies (n = 15, 1.5% of the total cohort; 198 POD cases total) were resolved through consensus discussion with a third senior neurologist. Inter-rater agreement was assessed using Cohen’s kappa (κ = 0.82, 95% CI 0.76–0.88), indicating excellent reliability.

Prior to the study, three researchers received professional training in identifying POD. Two researchers independently assessed and recorded cases of POD, with any discrepancies resolved through discussion or by the Senior Researcher’s determination, as needed.

### Statistical analysis

For descriptive statistics, categorical variables were summarized as percentages and compared between groups using chi-square tests. Continuous variables were expressed as mean ± standard deviation (SD) and analyzed using independent sample t-tests. To address missing data, we employed multiple imputation with chained equations (MICE), implemented using the R package ‘mice’, which generates less biased estimates than other methods.

The association between intraoperative CV-MAP and POD was investigated using logistic regression analysis. In the univariate logistic regression, we included variables with p-values < 0.05 in the multivariate logistic regression to adjust for potential confounders. Adjusted odds ratios (ORs) with 95% confidence intervals (CIs) were reported.

To minimize confounding effects, propensity score matching (PSM) was performed. Patients were matched in a 1:1 ratio using the nearest neighbor algorithm with a caliper width of 0.25 SD. Covariate balance was evaluated using standardized mean differences (SMDs), with SMD < 0.1 indicating adequate balance^35^. Subgroup analyses were conducted within the PSM cohort to further explore the relationship between CV-MAP and POD across different patient characteristics, such as ASA classification and comorbidities.

Additionally, CV-MAP was categorized into tertiles (T1: ≤ 7.56%, T2: 7.56–10.81%, T3: > 10.81%) to evaluate dose–response relationships. Univariate and multivariate logistic regression models were used to estimate the ORs for POD risk in each tertile, with T1 serving as the reference group. Restricted cubic spline (RCS) analysis was also performed to assess the nonlinear relationship between CV-MAP and POD.

All statistical analyses were performed using SPSS version 26 (IBM Corp., Armonk, NY, USA) and R version 4.0.3 (R Foundation for Statistical Computing, Vienna, Austria). A two-tailed p-value < 0.05 was considered statistically significant.

## Results

### Study population and baseline characteristics

**Fig. 1:**
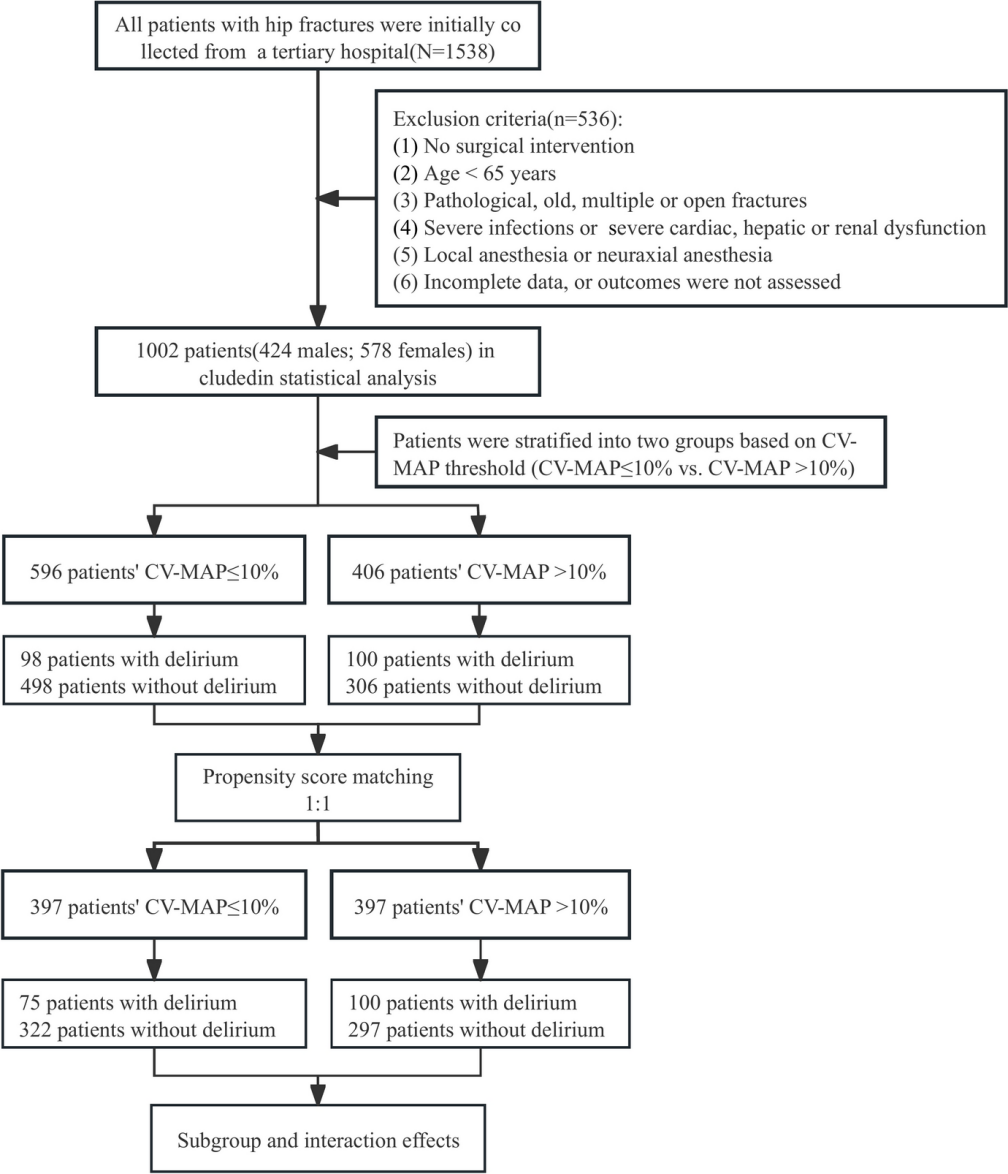
Flow diagram of enrollment.

The flowchart depicted in Fig. 1 outlines the step-by-step process of this study. It serves as a visual representation of the workflow, helping to clarify the sequence of activities and decision points involved. Following the enrollment workflow detailed in Fig. 1, Table 1 summarizes the baseline demographic and clinical characteristics of the 1,002 patients stratified by POD status, highlighting key differences between groups. Key findings include older age (84.38 years vs. 77.98 years, p < 0.001), higher prevalence of comorbidities (including hypertension, cardiovascular disease, cerebral infarction), and greater intraoperative CV-MAP in the POD group.

**Table 1:** Baseline demographic characteristics of patients with and without predicting postoperative delirium.

Variables	Total patients (n = 1002)	Groups		^†^P for trend
		Without delirium group (n = 804)	Delirium group (n = 198)	
Demographic				
Male gender (n, %)	424 (42.3)	350 (43.5)	74 (37.5)	0.116
Age, × year (mean, SD)	79.25 (9.70)	77.98 (9.49)	84.38 (8.83)	< 0.001
Smoking (n, %)	179 (17.9)	154 (19.2)	25 (12.5)	0.032
Alcohol (n, %)	124 (12.4)	102 (12.7)	22 (11.1)	0.546
Comorbidities				
Hypertension (n, %)	463 (46.2)	331 (41.2)	132 (66.7)	< 0.001
Diabetes (n, %)	111 (11.1)	87 (10.8)	24 (12.1)	0.602
COPD (n, %)	108 (10.8)	81 (10.1)	27 (13.6)	0.148
Cardiovascular disease (n, %)	281 (28.0)	204 (25.4)	77 (38.9)	< 0.001
Cerebral infarction (n, %)	238 (23.8)	151 (18.8)	87 (43.9)	< 0.001
Dementia (n, %)	57 (5.7)	35 (4.4)	22 (11.1)	< 0.001
Intracerebral hemorrhage (n, %)	59 (5.9)	31 (3.9)	28 (14.1)	< 0.001
Chronic liver disease (n, %)	48 (4.8)	37 (4.6)	11 (5.6)	0.574
Chronic kidney disease (n, %)	49 (4.9)	30 (3.7)	19 (9.6)	0.001
Tumor (n, %)	95 (9.5)	74 (9.2)	21 (10.6)	0.546
Chronic steroid use (n, %)	6 (0.6)	4 (0.5)	2 (1.0)	0.402
Operation				
Fracture type				
Femoral neck fracture (n, %)	559 (55.8)	457 (56.8)	102 (51.5)	0.012
Intertrochanteric fracture (n, %)	392 (39.1)	300 (37.3)	92 (46.5)	
Subtrochanteric fracture (n, %)	51 (5.1)	47 (5.8)	4 (2.0)	
Surgery type				
Total hip arthroplasty (n, %)	127 (12.7)	94 (11.7)	33 (16.7)	< 0.001
Hemiarthroplasty (n, %)	251 (25.0)	187 (23.3)	64 (32.3)	
Intramedullary nail fixation (n, %)	309 (30.8)	241 (30.0)	68 (34.3)	
Internal fixation with steel plate (n, %)	126 (126)	105 (13.1)	21 (10.6)	
Internal fixation with hollow nails (n, %)	189 (18.9)	177 (22.0)	12 (6.1)	
Intraoperative blood loss, × ml (mean, SD)	170.29 (145.83)	168.81 (151.87)	176.31 (118.33)	0.452
Intraoperative time, × hour (mean, SD)	1.62 (0.66)	1.61 (0.65)	1.63 (0.67)	0.732
Intraoperative CV-MAP, (mean, SD)	9.66 (4.23)	9.09 (3.24)	11.98 (6.45)	< 0.001
Transfusion (n, %)	157 (15.7)	115 (14.3)	42 (21.2)	0.017
Postoperative ICU (n, %)	67 (6.7)	53 (4.9)	10 (5.4)	0.939
Bedridden time, × day (mean, SD)	5.60 (3.53)	5.50 (3.40)	6.02 (4.01)	0.092
ASA classification				
I-II (n, %)	531 (53.0)	423 (52.6)	48 (24.2)	< 0.001
III-IV (n, %)	471 (47.0)	381 (47.4)	150 (75.8)	
Laboratory findings				
WBC count, × 10^9^/L (mean, SD)	8.66 (2.81)	8.64 (2.79)	8.76 (2.90)	0.609
NEU count, × 10^9^/L (mean, SD)	6.57 (2.72)	6.54 (2.71)	6.69 (2.73)	0.466
HGB level, × g/L (mean, SD)	120.30 (20.49)	122.30 (19.55)	112.21 (22.21)	< 0.001
K, × mmol/L (mean, SD)	4.00 (0.45)	3.99 (0.44)	4.03 (0.52)	0.296
Na, × mmol/L (mean, SD)	139.83 (4.81)	139.97 (4.92)	139.24 (4.30)	0.055
Blood glucose, × mmol/L (mean, SD)	5.84 (0.88)	5.80 (0.85)	6.01 (0.96)	0.005
Cr, × mmol/L (mean, SD)	69.87 (53.49)	69.16 (53.41)	72.75 (53.80)	0.401
Albumin, × g/L (mean, SD)	38.10 (4.66)	38.70 (4.44)	35.63 (4.73)	< 0.001
d-Dimer, × mg/L (mean, SD)	4.72 (4.86)	4.75 (4.92)	4.60 (4.62)	0.686

^†^P values are from Fisher’s exact test for continuous variables and from the chi-square test for categorical variables.

SD, Standard deviation; COPD, Chronic obstructive pulmonary disease; CV-MAP, the coefficient of variation in mean arterial pressure; ICU, Intensive Care Unit; ASA: the American Society of Anesthesiologists Physical Status Classification System; WBC, White blood cell; NEU, Neutrophil, HGB, hemoglobin;K, Potassium; Na, Sodium; RBC, red blood cell; WBC, White blood cell; Cr, Creatinine.

### Multivariate analysis for POD

The multivariate analysis identified several significant factors associated with POD in geriatric patients undergoing hip fracture surgery (Supplement eTable 1). Age (adjusted OR: 1.04, 95% CI 1.01–1.06, p = 0.007), hypertension (adjusted OR: 2.09, 95% CI 1.41–3.12, p = 0.002), stroke (adjusted OR: 1.91, 95% CI 1.28–2.85, p = 0.002), and intracerebral hemorrhage (adjusted OR: 3.34, 95% CI 1.75–6.38, p < 0.001) were strongly associated with increased POD risk. Patients with higher ASA classification (III-IV vs. I-II; adjusted OR: 1.60, 95% CI 1.04–2.46, p = 0.032) and elevated intraoperative CV-MAP (per 1% increase; adjusted OR: 1.14, 95% CI 1.09–1.19, p = 0.001) also exhibited a higher likelihood of POD. Lower albumin levels were inversely associated with POD risk(adjusted OR: 0.91, 95% CI 0.87–0.95, p < 0.001). These findings highlight potential risk factors for POD, suggesting areas for further investigation in mitigating its incidence.

### Propensity score matching

Table 2 presents a comparison of patient characteristics before and after propensity score matching (PSM) based on intraoperative blood pressure variability (CV-MAP ≤ 10% vs. > 10%). Before PSM, significant imbalances were observed in key variables such as age, comorbidities (e.g., cardiovascular disease, cerebral infarction), and surgical factors, highlighting differences between the two groups. After PSM, balance across covariates improved, as indicated by standardized mean differences (SMD < 0.1). The table underscores the rigorous matching process used to ensure comparability and reduce confounding in analyzing the impact of CV-MAP on postoperative outcomes.

**Table 2:** Patient characteristics before and after propensity score matching by intraoperative CV-MAP levels (normal ≤ 10% vs. high > 10%).

Variables	Before PSM			After PSM		
	CV-MAP ≤ 10% (n = 569)	CV-MAP > 10% (n = 406)	SMD	CV-MAP ≤ 10% (n = 397)	CV-MAP > 10% (n = 397)	SMD
Demographic						
Male gender (n, %)	111 (43.4)	395 (39.0)	0.089	95 (41.7)	90 (39.5)	0.045
Age, × year (mean, SD)	78.58 (9.53)	75.00 (13.00)	0.655	74.00 (17.00)	74.50 (14.00)	0.019
Smoking (n, %)	31 (12.1)	187 (18.4)	0.177	29 (12.7)	27 (11.8)	0.027
Alcohol (n, %)	22 (8.6)	126 (12.4)	0.125	21 (9.2)	23 (10.1)	0.030
Comorbidities						
Hypertension (n, %)	138 (53.9)	498 (49.10)	0.096	125 (54.8)	134 (58.8)	0.080
Diabetes (n, %)	50 (19.5)	241 (23.8)	0.103	47 (20.6)	53 (23.2)	0.063
COPD (n, %)	39 (15.2)	110 (10.8)	0.130	33 (14.5)	27 (11.8)	0.078
Cardiovascular disease (n, %)	94 (36.7)	296 (29.2)	0.160	80 (35.1)	76 (33.3)	0.037
Cerebral infarction (n, %)	91 (35.5)	239 (23.6)	0.264	74 (32.5)	73 (32.0)	0.009
Dementia (n, %)	13 (5.1)	35 (3.5)	0.080	10 (4.4)	7 (3.1)	0.069
Intracerebral hemorrhage (n, %)	13 (5.1)	55 (5.4)	0.015	13 (5.7)	8 (3.5)	0.105
Chronic liver disease (n, %)	17 (6.6)	41 (4.0)	0.116	15 (6.6)	14 (6.1)	0.018
Chronic kidney disease (n, %)	16 (6.3)	48 (4.7)	0.067	15 (6.6)	20 (8.8)	0.082
Tumor (n, %)	58 (9.7)	37 (9.1)	0.021	33 (8.3)	36 (9.1)	0.026
Chronic steroid use (n, %)	3 (0.5)	3 (0.7)	0.028	2 (0.5)	3 (0.8)	0.028
Operation						
Fracture type						
Femoral neck fracture (n, %)	91 (35.5)	588 (58.0)	0.410	86 (37.7)	85 (37.3)	0.036
Intertrochanteric fracture (n, %)	146 (57.0)	371 (36.6)		126 (55.3)	123 (53.9)	
Subtrochanteric fracture (n, %)	19 (7.4)	55 (5.4)		16 (7.0)	20 (8.8)	
Surgery type						
Total hip arthroplasty (n, %)	24 (9.4)	137 (13.5)	0.011	22 (9.6)	13 (5.7)	0.026
Hemiarthroplasty (n, %)	52 (20.3)	267 (26.3)		48 (21.1)	59 (25.9)	
Intramedullary nail fixation (n, %)	113 (44.1)	301 (29.7)		96 (42.1)	96 (42.1)	
Internal fixation with steel plate (n, %)	48 (18.8)	120 (11.8)		44 (19.3)	41 (18.0)	
Internal fixation with hollow nails (n, %)	19 (7.4)	189 (18.6)		18 (7.9)	19 (8.3)	
Intraoperative blood loss, × ml (mean, SD)	196.56 (158.95)	169.94 (150.94)	0.172	200.00 (200.00)	196.00 (200.00)	0.072
Intraoperative time, × hour (mean, SD)	196.56 (158.95)	169.94 (150.94)	0.172	200.00 (200.00)	196.00 (200.00)	0.072
Intraoperative CV-MAP, (mean, SD)	196.56 (158.95)	169.94 (150.94)	0.172	200.00 (200.00)	196.00 (200.00)	0.072
Transfusion (n, %)	64 (25.0)	146 (14.4)	0.269	55 (24.1)	61 (26.8)	0.060
Postoperative ICU (n, %)	26 (10.2)	37 (3.6)	0.258	19 (8.3)	22 (9.6)	0.046
Bedridden time, × day (mean, SD)	6.77 (5.04)	5.68 (3.70)	0.247	(3.00)	5.00 (3.25)	0.019
ASA classification						
I-II (n, %)	180 (70.3)	530 (52.3)	0.377	157 (68.9)	154 (67.5)	0.028
III–IV (n, %)	76 (29.7)	484 (47.7)		71 (31.1)	74 (32.5)	
Laboratory findings						
WBC count, × 10^9/L (mean, SD)	8.49 (3.24)	8.85 (2.75)	0.019	8.75 (2.95)	8.65 (2.76)	0.037
NEU count, × 10^9/L (mean, SD)	6.58 (2.77)	6.55 (2.64)	0.834	6.64 (2.88)	6.54 (2.65)	0.038
HGB level, × g/L (mean, SD)	106.38 (20.91)	123.19 (19.13)	0.839	125.00 (25.00)	122.50 (17.75)	0.031
K, × mmol/L (mean, SD)	3.99 (0.45)	4.02 (0.46)	0.069	4.00 (0.46)	4.02 (0.47)	0.044
Na, × mmol/L (mean, SD)	139.66 (3.50)	140.08 (6.25)	0.067	139.68 (3.60)	140.06 (6.31)	0.059
Blood glucose, × mmol/L (mean, SD)	6.81 (2.36)	6.95 (2.71)	0.058	38.00 (5.00)	38.00 (6.00)	0.087
Cr, × mmol/L (mean, SD)	8.12 (4.15)	7.21 (4.99)	0.198	69.73 (52.94)	71.83 (64.61)	0.011
Albumin , × g/L (mean, SD)	74.23 (48.61)	71.88 (67.80)	0.040	125.00 (25.00)	122.50 (17.75)	0.033
d-Dimer, × mg/L (mean, SD)	4.85 (4.60)	4.89 (5.14)	0.009	2.98 (4.43)	4.24 (7.45)	0.056

SD, Standard deviation; COPD, Chronic obstructive pulmonary disease; CV-MAP, the coefficient of variation in mean arterial pressure; ICU, Intensive Care Unit; ASA: the American Society of Anesthesiologists Physical Status Classification System; WBC, White blood cell; NEU, Neutrophil, HGB, hemoglobin;K, Potassium; Na, Sodium; RBC, red blood cell; WBC, White blood cell; Cr, Creatinine.

**Fig. 2:**
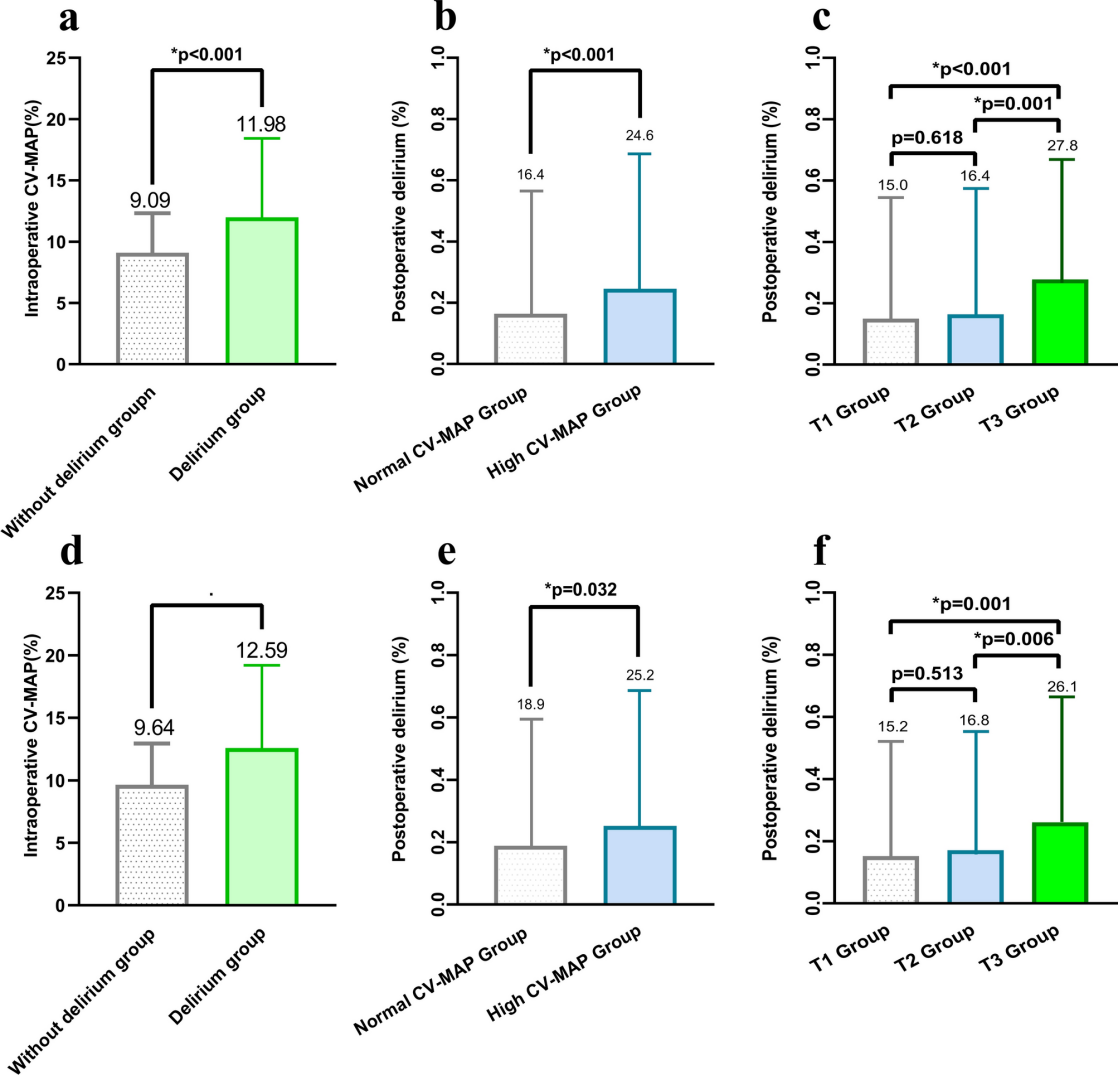
Relationship between different intraoperative CV-MAP level groups and POD rates in patients with hip fracture before and after PSM. (**a**) Mean and standard deviation of CV-MAP levels between the POD group and without POD group before PSM. (**b**) Comparison of POD rates before PSM based on intraoperative CV-MAP (CV-MAP ≤ 10% vs. > 10%). (**c**) Patients were categorized into 3 groups using tertiles, comparing POD rates among the 3 groups before PSM. (**d**) Mean and standard deviation of CV-MAP levels between the POD group and without POD group after PSM. (**e**) Comparison of POD rates after PSM based on intraoperative CV-MAP (CV-MAP ≤ 10% vs. > 10%). (**f**) Patients were categorized into 3 groups using tertiles, comparing POD rates among the 3 groups after PSM.

The y-axis of Fig. 2a shows the mean intraoperative CV-MAP before PSM, with the without delirium group averaging 9.09% and the delirium group averaging 11.98%, a statistically significant difference (*p < 0.001). Figure 2d presents the data after PSM, where the without delirium group had a CV-MAP of 9.64%, compared to 12.59% in the delirium group.

Table 3, Fig. 2b and e compares the incidence of POD before and after propensity score matching (PSM) based on intraoperative blood pressure variability (CV-MAP ≤ 10% vs. > 10%). Before PSM, the POD incidence was 16.44% in the CV-MAP ≤ 10% group versus 24.63% in the CV-MAP > 10% group (Fig. 2b and Table 3). After PSM, the difference persisted, with POD rates of 18.9% in the CV-MAP ≤ 10% group and 25.2% in the CV-MAP > 10% group (p = 0.032) (Fig. 2e and Table 3). These results demonstrate a consistent association between elevated CV-MAP and higher POD risk, even after adjustment for confounders.

**Table 3:** Comparison of the incidence of delirium before and after PSM based on intraoperative CV-MAP levels.

IntraoperativCV-MAP	No. (%) Cutoff value	Before PSM		*p-value	No. (%) Clinical cutoffs	After PSM		*p-value
		Without delirium	Delirium			Without delirium	Delirium	
Cutoff value	≤ 10% (596)	498 (83.56)	98 (16.44)	< 0.001	≤ 10% (397)	322 (81.1)	75 (18.9)	0.032
	> 10% (406)	306 (75.37)	100 (24.63)		> 10% (397)	297 (74.8)	100 (25.2)	

PSM, propensity scores matching; CV-MAP, the coefficient of variation in mean arterial pressure.

*p-value is from Chi-Squared Test to indicate significant differentiation (p < 0.05 means significant differentiation).

### Association between intraoperative blood pressure variability and POD

The association between intraoperative CV-MAP and POD in geriatric patients undergoing hip fracture surgery is comprehensively analyzed in Table 4. The table presents three models to evaluate this relationship: unadjusted analysis (Model 1), multivariable regression adjusted for confounding factors (Model 2), and propensity score-matched (PSM) analysis (Model 3).

**Table 4:** Unadjusted and adjusted association between Intraoperative CV-MAP levels and delirium.

Type	Intraoperative CV-MAP (%)	Model 1 (unadjusted OR)	p* trend 1	Model 2 (multivariable regression adjusted OR)	p* trend 2	Model 3 (PSM adjusted OR)	p* trend 3
Continuous	Per 1	1.16 (1.12–1.21)	< 0.001	1.14 (1.09–1.19)	0.001	NA	NA
Cutoff value	≤ 10%	1 [Reference]	0.001	1 [Reference]	0.005	1 [Reference]	0.033
	> 10%	1.66 (1.21–2.27)		1.67 (1.17–2.39)		1.45 (1.03–2.03)	
Tertiles	T1 (≤ 7.56%)	1 [Reference]	NA	1 [Reference]	NA	1 [Reference]	NA
	T2 (7.56–10.81%)	1.11 (0.73–1.69)	0.619	1.17 (0.73–1.86)	0.512	1.15 (0.75–1.77)	0.513
	T3 (> 10.81%	2.18 (1.49–3.21)	< 0.001	2.22 (1.44–3.45)	< 0.001	1.97 (1.31–2.95)	0.001

CI, confidence interval; OR, odds ratio; PSM, propensity scores matching; CV-MAP, the coefficient of variation in mean arterial pressure.

*P for trend; NA#, Not applicable.

When treated as a continuous variable, CV-MAP was significantly associated with an increased risk of POD. In Model 1, each 1% increase in CV-MAP was associated with a 16% higher risk of POD (odds ratio [OR]: 1.16, 95% CI 1.12–1.21, p < 0.001). This association persisted after adjusting for potential confounders, including age, hypertension, stroke, intracerebral hemorrhage, ASA classification, Surgery type and albumin, (adjusted OR: 1.14, 95% CI 1.09–1.19, p = 0.001). Due to sample size limitations, PSM-adjusted analysis for the continuous variable was not performed.

Patients were further categorized into two groups based on a CV-MAP cutoff of 10%. Compared to those with CV-MAP ≤ 10%, patients with CV-MAP > 10% exhibited a significantly higher risk of POD in all models. The unadjusted analysis (Model 1) indicated an OR of 1.66 (95% CI 1.21–2.27, p = 0.001), which increased to 1.67 (95% CI 1.17–2.39, p = 0.005) after adjusting for confounders in Model 2. PSM analysis in Model 3 further confirmed this finding, with an OR of 1.45 (95% CI 1.03–2.03, p = 0.033).

Additionally, Patients were divided into three CV-MAP tertiles: T1 (≤ 7.56%), T2 (7.56–10.81%), and T3 (> 10.81%). T1 was the reference group. Although T2 showed no significant POD risk difference compared to T1, T3 consistently had higher risk. In the unadjusted analysis, T3 was associated with an OR of 2.18 (95% CI 1.49–3.21, p < 0.001). The association remained significant after multivariable adjustment (adjusted OR: 2.22, 95% CI 1.44–3.45, p < 0.001) and PSM analysis (adjusted OR: 1.97, 95% CI 1.31–2.95, p = 0.001). These findings emphasize a dose–response relationship, with a pronounced increase in POD risk observed in the highest tertile of CV-MAP.

Trends remained consistent before and after PSM, with T3 consistently showing a higher POD incidence (Fig. 2c and f). Before PSM, the incidence was 15.0% in T1, 16.4% in T2, and 27.8% in T3. After PSM, the incidence was 15.2% in T1, 16.8% in T2, and 26.1% in T3. These findings suggest that higher MAP variability (CV-MAP) in the T3 group is associated with an increased risk of POD, and this association persists even after adjusting for confounders, highlighting CV-MAP as a potential key risk factor for POD.

**Fig. 3:**
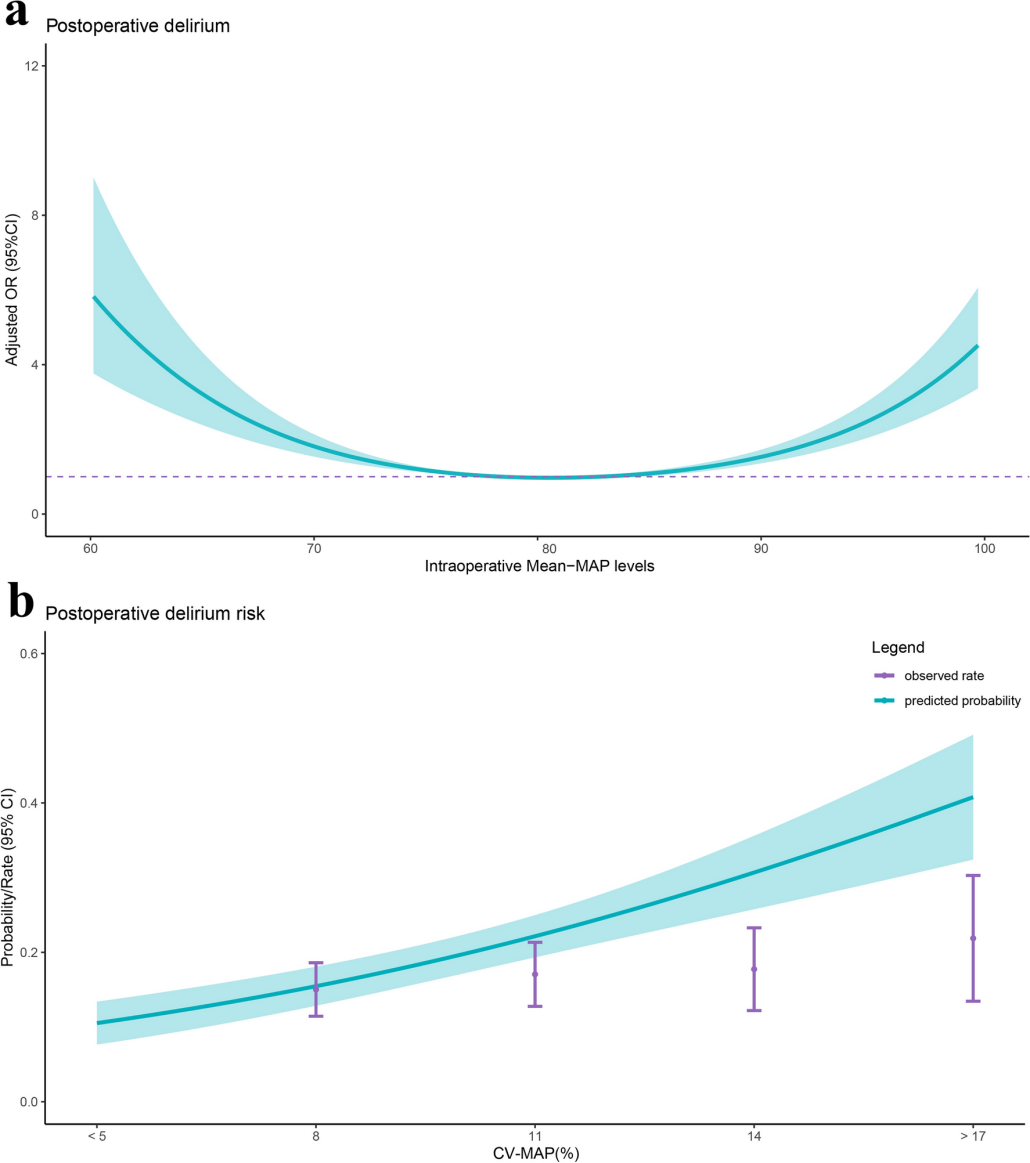
Relationship between intraoperative Mean − MAP levels and POD in patients with hip fracture. (**a**) Adjusted odds ratios (ORs) for postoperative delirium across intraoperative mean-MAP levels—showing a U-shaped pattern—include ORs and 95% confidence intervals (CIs) for 10 mmHg deviation from the reference value of intraoperative Mean − MAP levels. (**b**) Predicted probabilities and the observed rate of POD based on CV-MAP.

Figure 3a illustrates the relationship between POD and intraoperative MAP. The y-axis represents the adjusted OR (with 95% CI), while the x-axis shows intraoperative MAP levels. The curve exhibits a U-shaped pattern, indicating that both low MAP values (around 60 mmHg) and high MAP values (around 100 mmHg) are associated with an increased risk of POD. In contrast, MAP values near 80 mmHg are associated with the lowest risk. This illustrates the relationship and the trend of MAP’s impact on POD risk.

Figure 3b presents the restricted cubic spline (RCS) curve for the risk of POD. The y-axis represents the probability/odds (with 95% confidence intervals), and the x-axis shows CV-MAP. The light green area illustrates the predicted probability of POD, while the purple vertical lines represent the observed incidence rates of POD. Both the predicted probability and observed incidence increase as CV-MAP rises, highlighting the positive association between intraoperative blood pressure variability and POD risk.

**Fig. 4:**
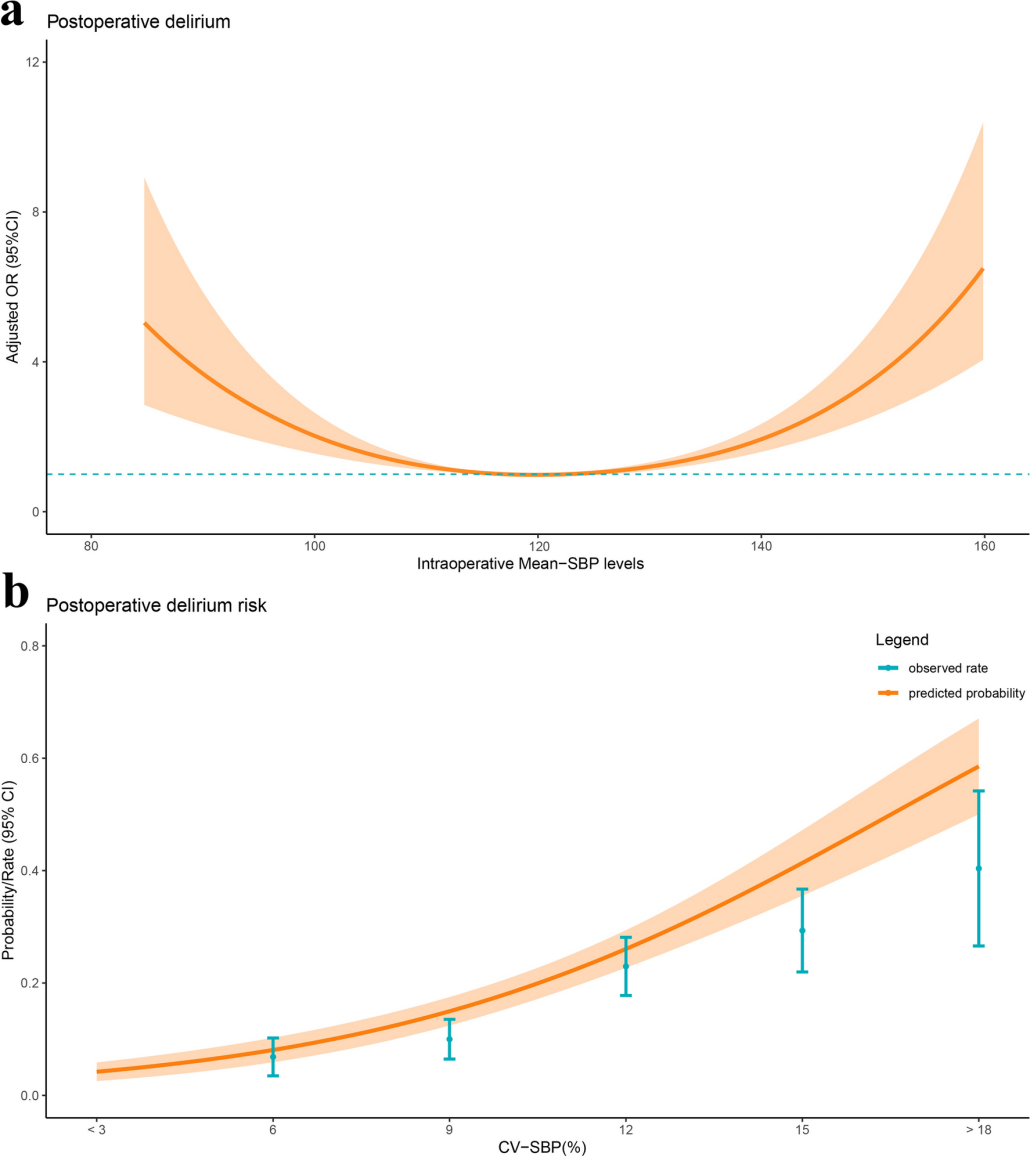
Relationship between intraoperative Mean − SBP levels and POD in patients with hip fracture. (**a**) Adjusted odds ratios (ORs) and 95% confidence intervals (CIs) are shown for 10 mmHg deviation away from the reference value of intraoperative Mean − SBP levels. (**b**) Predicted probabilities and the observed rate of POD based on CV-SBP.

Similarly, the relationship between POD and intraoperative mean systolic blood pressure (mean-SBP) levels also follows a U-shaped curve (Fig. 4a). This indicates that both excessively high and low intraoperative mean-SBP levels are associated with higher adjusted odds ratios for POD, whereas an intermediate SBP level (around 120 mmHg) corresponds to a relatively lower odds ratio and reduced risk. Additionally, as the coefficient of variation in systolic blood pressure increases, both the predicted probability and observed occurrence of POD rise, suggesting that greater systolic CV-MAP is associated with a higher risk of POD (Fig. 4b).

For diastolic blood pressure (DBP), the relationship between intraoperative mean DBP levels and POD risk approximates a U-shaped curve (eFig. 1a). However, the risk remains relatively stable at lower DBP levels and increases sharply once a certain threshold (around 70 mmHg) is exceeded. This indicates that systolic blood pressure (SBP) has a relatively broader “safe range” concerning POD risk, while the “safe range” for DBP may be narrower, with a more pronounced risk increase beyond its upper limit. In contrast, the impact of the coefficient of variation in DBP on POD risk is relatively minor (eFig. 1b) , showing overall gradual changes and a narrower fluctuation range. These findings indicate that SBP stability is likely more critical for reducing POD risk, while DBP stability, although relevant, plays a comparatively weaker role.

**Fig. 5:**
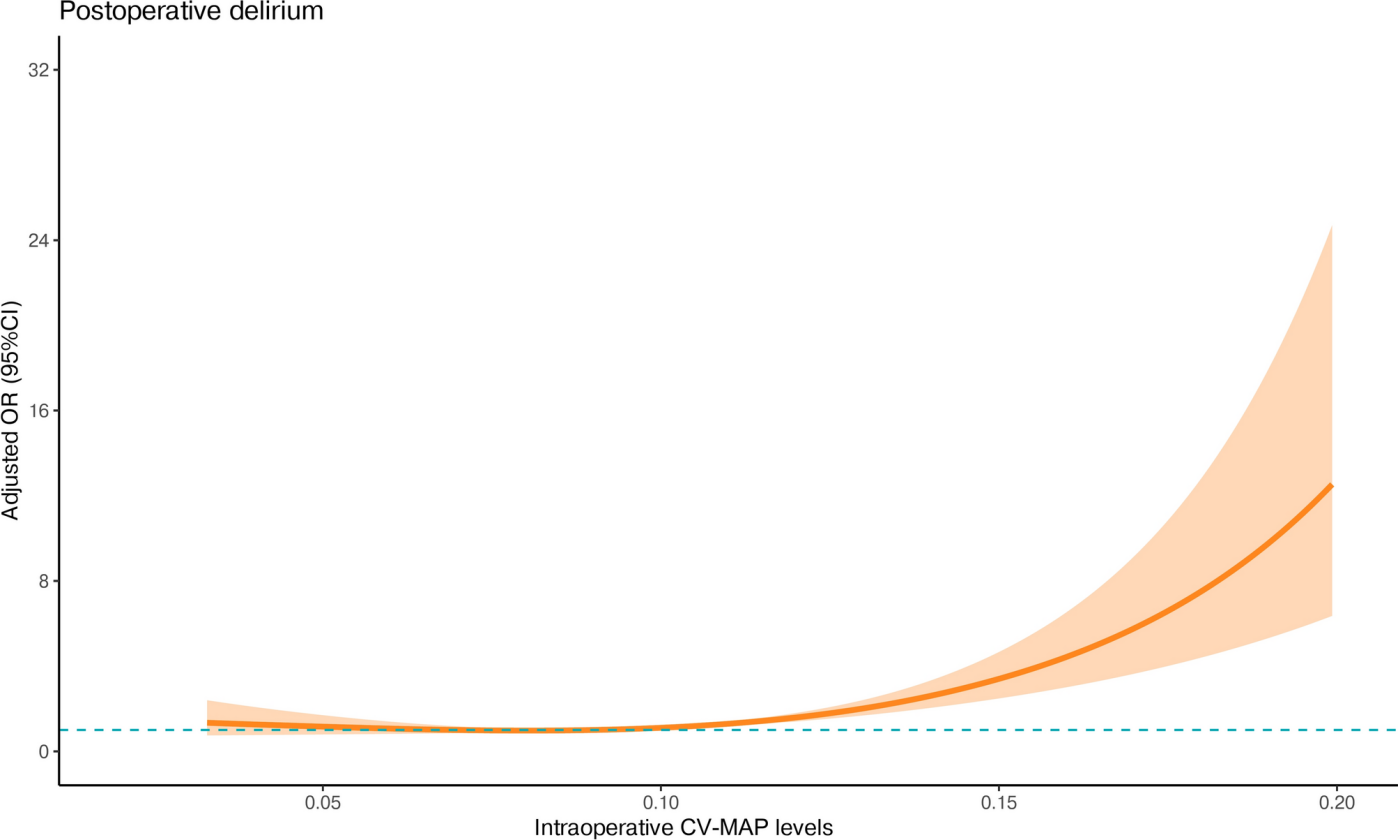
Adjusted odds ratios (ORs) and 95% confidence intervals (CIs) are shown for each 5% deviation away from the reference value of intraoperative CV-MAP level. A logistic regression model was first fitted, incorporating restricted cubic spline transformations for CV-MAP to capture its non-linear association with postoperative delirium. Based on the model’s coefficients and spline transformations, predicted adjusted odds ratios (ORs) and their 95% confidence intervals (CIs) were calculated for different CV-MAP values. These results were then visualized to illustrate the relationship between intraoperative CV-MAP levels and adjusted ORs for POD.

Predicted probabilities and observed odds ratios (ORs) for POD based on intraoperative CV-MAP levels (Fig. 5) show a heightened risk of delirium with increasing intraoperative CV-MAP levels.

Overall, these results highlight a robust and consistent association between elevated intraoperative blood pressure variability and POD risk. The findings underscore the importance of individualized intraoperative blood pressure management to mitigate the risk of POD, particularly in patients with a CV-MAP exceeding 10% or in the upper tertile.

### Interaction analysis

**Fig. 6:**
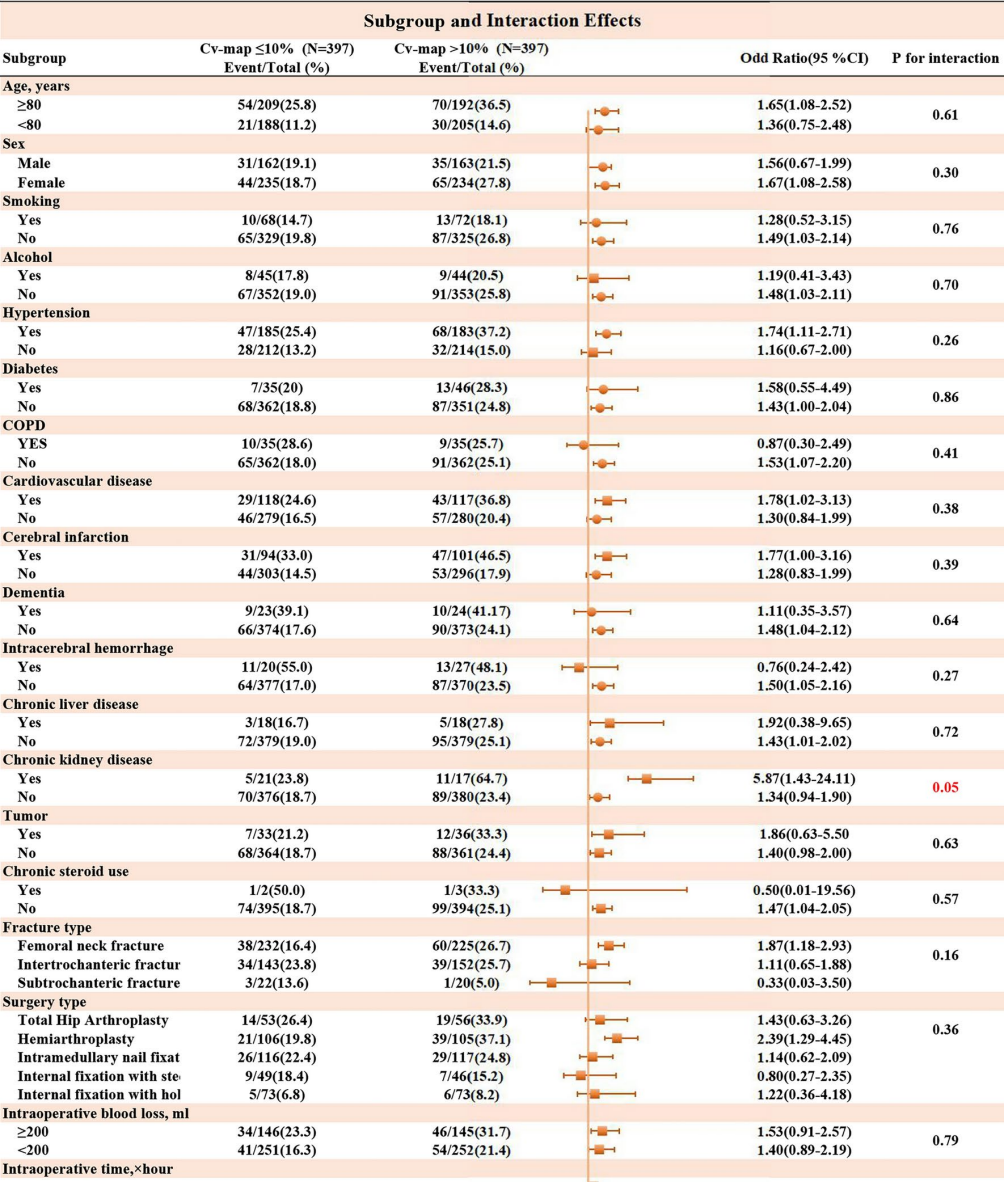

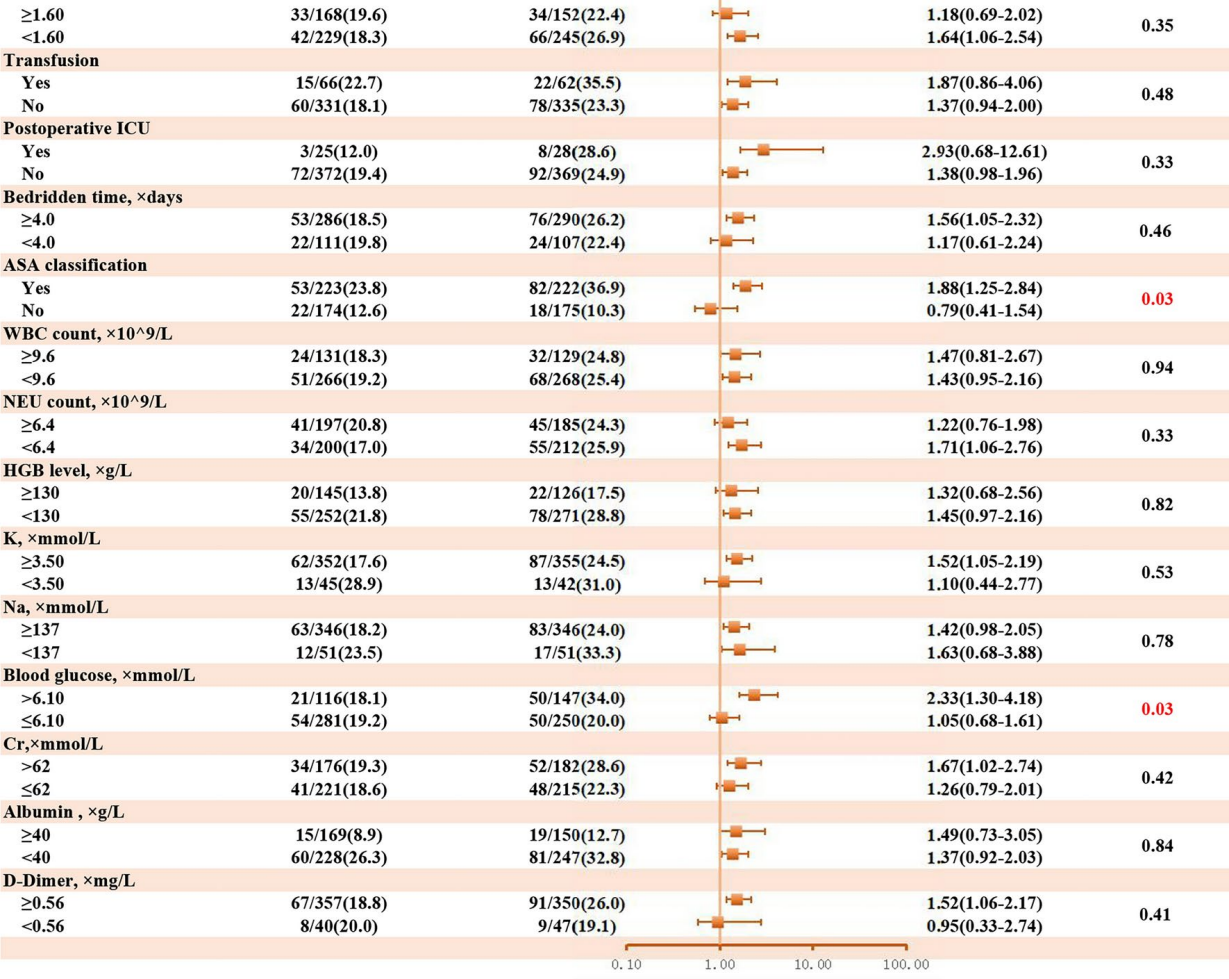
Subgroup analysis of association CV-MAP and POD after propensity score matching.

Building on the observed dose–response relationship between intraoperative BPV and POD risk, we further investigated whether patient-specific factors modified this association through subgroup interaction analyses (Fig. 6).A notable interaction was observed for chronic kidney disease (CKD). Patients with CKD demonstrated a markedly higher risk of POD with CV-MAP > 10% (OR: 5.87, 95% CI 1.43–24.11, p for interaction = 0.05), whereas the association was weaker in patients without CKD (OR: 1.34, 95% CI 0.94–1.90). Patients with ASA III-IV classification had a stronger association between CV-MAP > 10% and POD (OR: 1.88, 95% CI 1.25–2.84), compared to those with ASA I-II classification, which showed no significant association (OR: 0.79, 95% CI 0.41–1.54). This interaction was statistically significant (p = 0.03). Among patients with blood glucose > 6.10 mmol/L, CV-MAP > 10% significantly increased the risk of POD (OR: 2.33, 95% CI 1.30–4.18), while no significant association was found in those with blood glucose ≤ 6.10 mmol/L (OR: 1.05, 95% CI 0.68–1.61). The interaction test was significant (p = 0.03).

## Discussion

In this study, we examined the association between intraoperative BPV and POD in geriatric patients undergoing hip fracture surgery. Our findings indicate that increased BPV is significantly associated with a higher incidence of POD, underscoring the clinical relevance of monitoring intraoperative hemodynamic stability as a potential modifiable factor in this vulnerable population.

Our results align with previous research highlighting the impact of intraoperative hemodynamic fluctuations on postoperative outcomes. A study by Wang et al. found a J-shaped association between absolute levels of mean surgery MAP (msMAP) and PD risk. Higher msMAP (≥ 80 mmHg) was associated with an increased PD risk (OR = 2.28 per 10 mmHg increase), while lower msMAP (< 80 mmHg) was linked to a lower PD risk (OR = 0.19 per 10 mmHg increase) in elderly hip fracture patients^36^. No significant relationship was found between PD risk and the percent change from baseline in msMAP. The study concluded that both very high and very low msMAP levels increased the risk of PD in elderly hip fracture patients. Unlike Wang et al., who measured BPV via absolute hypotension thresholds, our use of CV-MAP accounts for relative variability, enabling direct comparison across heterogeneous populations. While Hirsch et al. (2021) observed no significant association between intraoperative hypotension (relative or absolute) and POD in non-cardiac surgery patients, their findings align partially with ours: both studies highlight blood pressure variability (BPV) as a critical risk factor ^20^. The apparent discrepancy regarding hypotension may stem from differences in patient populations (e.g., hip fracture vs. general non-cardiac surgery), thresholds for defining hypotension (e.g., MAP < 50 mmHg vs. alternative metrics), or methodologies for quantifying BPV. Notably, Hirsch et al. focused on short-term variability, whereas our study employed the coefficient of variation (CV-MAP), which normalizes variability to mean pressure, potentially enhancing sensitivity in elderly cohorts. These distinctions underscore the need for standardized definitions and context-specific risk assessments in perioperative care. A study by Zhang found that MAPV greater than 2.17 was associated with a higher risk of POD in elderly patients after hip fracture surgery^37^. The multivariate analysis showed that higher MAPV increased the risk of POD (OR: 2.379, P < 0.001). After adjusting for other factors using propensity score matching, this association remained significant (OR: 2.851, P < 0.001). Compared to this study, in our research, intraoperative BPV was quantified using CV_MAP, offering a more standardized and precise measure than MAPV. This approach enables a better assessment of relative BP fluctuations, making it more applicable to diverse patient populations with varying baseline blood pressure levels. A study by Shen et al. concluded that maintaining stable blood pressure postoperatively could help reduce the risk of POD in cardiac surgery patients^38^. However, in a study included 2,352 patients by Technologies on patients over 70 undergoing elective non-cardiac surgery, no association was found between intraoperative hypotension and POD^39^.

*From a treatment perspective, our findings highlight the need for intraoperative hemodynamic stabilization using strategies such as continuous invasive monitoring, titrated vasopressors, and regional anesthesia to minimize BPV. Preoperative optimization of hypertension and hyperglycemia further enhances cerebrovascular resilience, reducing vulnerability to BPV-induced insult.* Additionally, preoperative BPV may reflect underlying vascular dysfunction and should be addressed through antihypertensive therapy and lifestyle modifications. Stabilizing preoperative blood pressure reduces intraoperative variability, thereby lowering POD risk.

The pathophysiological mechanisms linking intraoperative BPV to POD are complex and likely multifactorial. One hypothesized pathway involves disruptions in cerebral autoregulation—a mechanism thought to maintain stable cerebral blood flow across systemic blood pressure ranges. When BPV exceeds hypothesized autoregulatory thresholds, transient hypoperfusion may lead to ischemic injury or neuronal apoptosis, while hyperperfusion could result in oxidative stress or endothelial dysfunction^20,40–43^. Both scenarios contribute to neuroinflammation, a key driver of delirium pathogenesis.

Additionally, BPV may exacerbate systemic inflammation, another recognized factor in POD development^44,45^. Fluctuations in blood pressure have been associated with increased levels of pro-inflammatory cytokines, which can cross the blood–brain barrier and activate microglia^46,47^. This neuroinflammatory cascade is thought to disrupt synaptic connectivity and impair neurotransmitter systems, including cholinergic and dopaminergic pathways, leading to the cognitive and behavioral manifestations of delirium^48,49^.

The multivariate analysis identifies several significant factors associated with POD in geriatric patients undergoing hip fracture surgery. These risk factors likely contribute to POD through distinct yet interrelated pathophysiological mechanisms. Advanced age (adjusted OR: 1.04) remained a significant predictor of POD, consistent with prior studies linking aging to reduced cognitive reserve and pre-existing neurodegeneration (e.g., Alzheimer’s or vascular dementia), which impair neuronal plasticity and resilience to perioperative stressors. Aging also exacerbates susceptibility to cerebral hypoperfusion and neuroinflammation, both implicated in delirium pathogenesis. These factors collectively lower the threshold for delirium in elderly patients, even in the absence of overt preoperative cognitive impairment^50^. Hypertension (adjusted OR: 2.09) was strongly associated with POD, likely due to chronic vascular remodeling and reduced cerebral autoregulatory capacity^51^. This aligns with evidence that hypertensive patients are more vulnerable to perioperative hemodynamic fluctuations, which can exacerbate brain injury. A history of stroke (adjusted OR: 1.91) and intracerebral hemorrhage (adjusted OR: 3.34) significantly increased POD risk, reflecting the role of preexisting cerebrovascular damage in lowering the brain’s resilience to perioperative stressors^52,53^. Higher ASA classification (III-IV vs. I-II; adjusted OR: 1.60) indicates greater systemic disease burden, which amplifies vulnerability to POD^50^. Lower albumin levels (adjusted OR: 0.91) were inversely associated with POD, reflecting the role of nutritional status and systemic inflammation in delirium risk^54,55^. The identified factors—age, hypertension, prior cerebrovascular events, ASA classification, intraoperative BPV, and albumin levels—highlight the multifactorial nature of POD and underscore the importance of individualized perioperative care. Addressing these modifiable risks can significantly improve outcomes in geriatric patients undergoing hip fracture surgery.

Our findings suggest that MAP values around 80 mmHg were associated with a lower risk of POD, though further studies are needed to establish clinical guidelines. This indicates that within this range, cerebral blood flow remains stable, avoiding both hypoperfusion and damage associated with excessive pressure. For systolic blood pressure (SBP), a broader “safe range” was observed, likely due to the brain’s autoregulation of blood flow relying primarily on SBP. Maintaining SBP around 120 mmHg appeared to support stable cerebral perfusion. In contrast, diastolic blood pressure (DBP) demonstrated a narrower "safe range," with greater sensitivity to fluctuations. Elevated DBP may increase peripheral vascular resistance, leading to cardiac strain and cerebral perfusion instability, while low DBP could compromise coronary perfusion, indirectly affecting cerebral blood flow. Notably, low diastolic blood pressure (DBP) in elderly patients is frequently associated with arterial stiffness, a hallmark of aging-related vascular remodeling, which may influence these findings.

Interestingly, this study found that intraoperative systolic BPV and MAP variability (MAPV) are significantly associated with POD, whereas diastolic BPV is not. This finding underscores the differing physiological roles of blood pressure components in cerebral autoregulation and POD risk.

SBP and MAP directly influence cerebral perfusion pressure, which is tightly regulated by autoregulatory mechanisms^56^. Large fluctuations in these parameters can lead to hypoperfusion or hyperperfusion, causing neuronal injury and neuroinflammation, key contributors to POD^57^. DBP, reflecting vascular tone, has a lesser role in acute changes in cerebral perfusion^58^. SBP and MAP are more dynamic markers of intraoperative stress, making their variability more predictive of adverse outcomes compared to DBP.

The observed interactions suggest that chronic kidney disease (CKD), higher ASA classification (III-IV), and elevated blood glucose levels (> 6.10 mmol/L) may amplify the effects of intraoperative BPV on POD. CKD is associated with impaired vascular autoregulation and heightened neuroinflammation, making the brain more vulnerable to perfusion fluctuations^59^. The stronger association between BPV and POD in CKD patients may be attributed to uremic toxins, such as indoxyl sulfate and p-cresyl sulfate, which accumulate in renal dysfunction^60,61^. These toxins disrupt the blood–brain barrier, induce neuroinflammation, and impair neurotransmitter systems, exacerbating vulnerability to delirium. Higher ASA classifications reflect reduced physiological reserve, increasing susceptibility to hemodynamic instability and cerebral injury^62–64^. Hyperglycemia exacerbates systemic inflammation, oxidative stress, and blood–brain barrier dysfunction, further amplifying the risk of neuroinflammation and neuronal damage^65^. These mechanisms highlight the importance of individualized perioperative management to mitigate POD risk in these high-risk subgroups.

The identification of BPV as an independent risk factor for POD has several practical implications. First, it reinforces the importance of intraoperative hemodynamic monitoring and control in geriatric surgical patients. Continuous blood pressure monitoring, ideally with invasive arterial lines, enables real-time detection and management of significant fluctuations. Anesthetic techniques and pharmacologic interventions should be tailored to minimize BPV while avoiding prolonged hypotension, which has its own set of adverse effects. Second, our findings suggest that preoperative optimization of cerebrovascular and systemic health may enhance resilience against BPV-induced insults. Interventions such as blood pressure stabilization, correction of anemia, and nutritional support could improve autoregulatory capacity and reduce inflammation, thereby lowering POD risk. Third, the dose–response relationship between BPV and POD risk suggests that even modest reductions in variability could yield significant clinical benefits.

The strengths of this study include its large sample size, robust statistical methods, and focus on a clinically relevant and vulnerable population. By employing PSM, we minimized confounding and ensured balanced comparison groups, enhancing the validity of our findings. While PSM improves the robustness of our findings, residual confounding remains a possibility, as unmeasured variables such as baseline frailty and cognitive status were not accounted for. Additionally, the use of restricted cubic spline analysis allowed us to explore nonlinear relationships, providing nuanced insights into the dose–response effects of BPV. Furthermore, our study extends the understanding of BPV’s impact by focusing on a geriatric population characterized by heightened vulnerability to cerebrovascular and cognitive insults. Compared to younger cohorts, older adults have reduced cerebrovascular reserve, increased prevalence of comorbidities, and greater susceptibility to neuroinflammatory processes, making BPV a particularly relevant concern in this group. By addressing these population-specific risks, our findings contribute to the growing body of evidence emphasizing the importance of tailored perioperative management strategies for elderly patients.

However, several limitations warrant consideration. The retrospective design precludes causal inference, and unmeasured confounding cannot be entirely excluded despite PSM. Our reliance on single-center data may limit generalizability, and variations in surgical and anesthetic practices across institutions could influence outcomes. Potential selection bias that may arise from single-center design. Furthermore, while CV-MAP provides a standardized measure of BPV, it may not capture all aspects of hemodynamic instability. *Third, while propensity score matching adjusted for numerous covariates, unmeasured confounders—such as baseline frailty (assessed *via* tools like the Clinical Frailty Scale) and preoperative cognitive function—may have influenced our findings. Frailty and cognitive impairment are well-established risk factors for POD but were not systematically documented in our dataset, potentially introducing residual confounding.*

Building on our findings, prospective studies are needed to establish causal relationships and refine BPV management strategies. Randomized controlled trials comparing different hemodynamic targets and variability thresholds could provide high-quality evidence to inform clinical guidelines. Additionally, research into the underlying mechanisms of BPV’s effects on the aging brain, including advanced neuroimaging and biomarker studies, could enhance our understanding of delirium pathophysiology and identify novel therapeutic targets.

## Conclusions

This study demonstrates that elevated intraoperative BPV independently increases POD risk in elderly hip fracture patients, particularly among high-risk subgroups (e.g., CKD, ASA III-IV). Our findings underscore the critical need for individualized hemodynamic management, including continuous intraoperative monitoring and targeted interventions to stabilize cerebral perfusion. Clinically, preoperative optimization of comorbidities and nutritional status may further mitigate risk. Future research must prioritize interventional trials—such as randomized controlled studies comparing BPV thresholds or neuroprotective strategies—to establish causal relationships and refine guidelines. These efforts will advance perioperative care, ultimately improving outcomes for this vulnerable population.

## Supplementary Information

Supplementary Information.

## Data Availability

All the data used and analyzed during the current study are available from the corresponding author upon reasonable request.
