# Supplementary material for: Correlation of intraoperative blood pressure variability and postoperative delirium in elderly hip fracture surgery
Source: Sci Rep. 2025 Apr 29;15:15007. doi: 10.1038/s41598-025-00019-0 (PMC12041256; doi:10.1038/s41598-025-00019-0)
Supplement: Supplementary file 1 — Supplementary Information. [file 41598_2025_19_MOESM1_ESM.docx]

**Supplement**

**eTable1 Multivariate Analysis for postoperative delirium.**

**eFigure 1 Relationship between intraoperative Mean−SBP levels and POD in patients with hip fracture. (a) Adjusted odds ratios (ORs) and 95% confidence intervals (CIs) are shown for 10 mmHg deviation away from the reference value of intraoperative Mean− DBP levels. (b) Predicted probabilities and the observed rate of POD based on CV-DBP.**

**Table 1 Multivariate Analysis for postoperative delirium**

| Variables | Univariate | | | Multivariate | | |
| --- | --- | --- | --- | --- | --- | --- |
|  | OR | 95%CI | p-value | OR | 95%CI | p-value |
| Demographic |  |  |  |  |  |  |
| Male gender | 0.77 | 0.56-1.07 | 0.117 | <NA> | <NA> | <NA> |
| Age, × year | 1.07 | 1.06-1.09 | <0.001 | 1.04 | 1.01-1.06 | 0.007 |
| Smoking | 0.61 | 0.39-0.96 | 0.033 | 1.10 | 0.63-1.90 | 0.743 |
| Alcohol | 0.86 | 0.53-1.40 | 0.547 | <NA> | <NA> | <NA> |
| Comorbidities |  |  |  |  |  |  |
| Hypertension | 2.86 | 2.06-3.96 | <0.001 | 2.09 | 1.41-3.12 | 0.002 |
| Diabetes | 1.14 | 0.70-1.84 | 0.602 | <NA> | <NA> | <NA> |
| COPD | 1.41 | 0.88-2.25 | 0.149 | <NA> | <NA> | <NA> |
| Cardiovascular disease | 1.87 | 1.35-2.60 | <0.001 | 0.94 | 0.63-1.41 | 0.770 |
| Stroke | 3.39 | 2.43-4.72 | <0.001 | 1.91 | 1.28-2.85 | 0.002 |
| Dementia(n, %) | 2.75 | 1.57-4.80 | <0.001 | 1.55 | 0.79-3.03 | 0.200 |
| Intracerebral hemorrhage | 4.11 | 2.40-7.03 | <0.001 | 3.34 | 1.75-6.38 | <0.001 |
| Chronic liver disease | 1.22 | 0.61-2.44 | 0.574 | <NA> | <NA> | <NA> |
| Chronic kidney disease | 2.74 | 1.51-4.98 | <0.001 | 1.98 | 0.99-3.95 | 0.052 |
| Tumor | 1.17 | 0.70-1.95 | 0.547 | <NA> | <NA> | <NA> |
| Chronic steroid use | 2.04 | 0.37-11.22 | 0.412 | <NA> | <NA> | <NA> |
| Operation |  |  |  |  |  |  |
| Fracture type |  |  |  |  |  |  |
| Femoral neck fracture | 1.00(Reference) | <NA> | <NA> | 1.00(Reference) | <NA> | <NA> |
| Intertrochanteric fracture | 3.60 | 1.27-10.26 | 0.016 | 2.41 | 0.60-9.66 | 0.215 |
| Subtrochanteric fracture | 2.62 | 0.92-7.44 | 0.070 | 1.56 | 0.28-8.57 | 0.611 |
| Surgery type |  |  |  |  |  |  |
| Total Hip Arthroplasty | 1.00(Reference) | - | - | 1.00(Reference) | - | - |
| Hemiarthroplasty | 0.97 | 0.60-1.59 | 0.919 | 0.42 | 0.23-0.76 | 0.005 |
| Intramedullary nail fixation | 0.80 | 0.50-1.30 | 0.372 | 0.22 | 0.07-0.74 | 0.015 |
| Internal fixation with steel plate | 0.57 | 0.31-1.05 | 0.072 | 0.29 | 0.08-1.07 | 0.063 |
| Internal fixation with hollow nails | 0.19 | 0.10-0.39 | 0.001 | 0.30 | 0.13-0.69 | 0.004 |
| Intraoperative blood loss | 1.00 | 1.00-1.00 | 0.517 | <NA> | <NA> | <NA> |
| Intraoperative time | 1.04 | 0.82-1.32 | 0.727 | <NA> | <NA> | <NA> |
| Intraoperative CV-MAP (Per 1%) | 1.16 | 1.12-1.21 | <0.001 | 1.14 | 1.09-1.19 | 0.001 |
| Transfusion | 1.61 | 1.09-2.39 | 0.017 | 0.97 | 0.58-1.60 | 0.891 |
| Postoperative ICU | 0.98 | 0.52-1.83 | 0.939 | <NA> | <NA> | <NA> |
| Bedridden time | 1.04 | 1.00-1.08 | 0.064 | <NA> | <NA> | <NA> |
| ASA classification |  |  |  |  |  |  |
| Ⅰ-Ⅱ | 1.00(Reference) | - | - | 1.00(Reference) | - | - |
| Ⅲ-Ⅳ | 3.47 | 2.44-4.94 | <0.001 | 1.60 | 1.04-2.46 | 0.032 |
| Laboratory findings |  |  |  |  |  |  |
| WBC count | 1.01 | 0.96-1.07 | 0.600 | <NA> | <NA> | <NA> |
| NEU count | 1.02 | 0.97-1.08 | 0.463 | <NA> | <NA> | <NA> |
| HGB level | 0.98 | 0.97-0.98 | <0.001 | 0.99 | 0.98-1.00 | 0.076 |
| K | 1.22 | 0.87-1.72 | 0.244 | <NA> | <NA> | <NA> |
| Na | 0.96 | 0.92-0.99 | 0.032 | 0.99 | 0.96-1.03 | 0.754 |
| Blood glucose | 1.31 | 1.10-1.57 | 0.003 | 1.16 | 0.94-1.43 | 0.166 |
| Cr | 1.00 | 1.00-1.00 | 0.404 | <NA> | <NA> | <NA> |
| Albumin | 0.087 | 0.83-0.90 | <0.001 | 0.91 | 0.87-0.95 | <0.001 |
| D-Dimer | 0.99 | 0.96-1.03 | 0.697 | <NA> | <NA> | <NA> |

Abbreviations: COPD, Chronic obstructive pulmonary disease; CV-MAP, the coefficient of variation in mean arterial pressure; ICU, Intensive Care Unit; ASA: the American Society of Anesthesiologists Physical Status Classification System; WBC, White blood cell; NEU, Neutrophil, HGB, hemoglobin;K, Potassium; Na, Sodium; RBC, red blood cell; WBC, White blood cell; Cr, Creatinine.


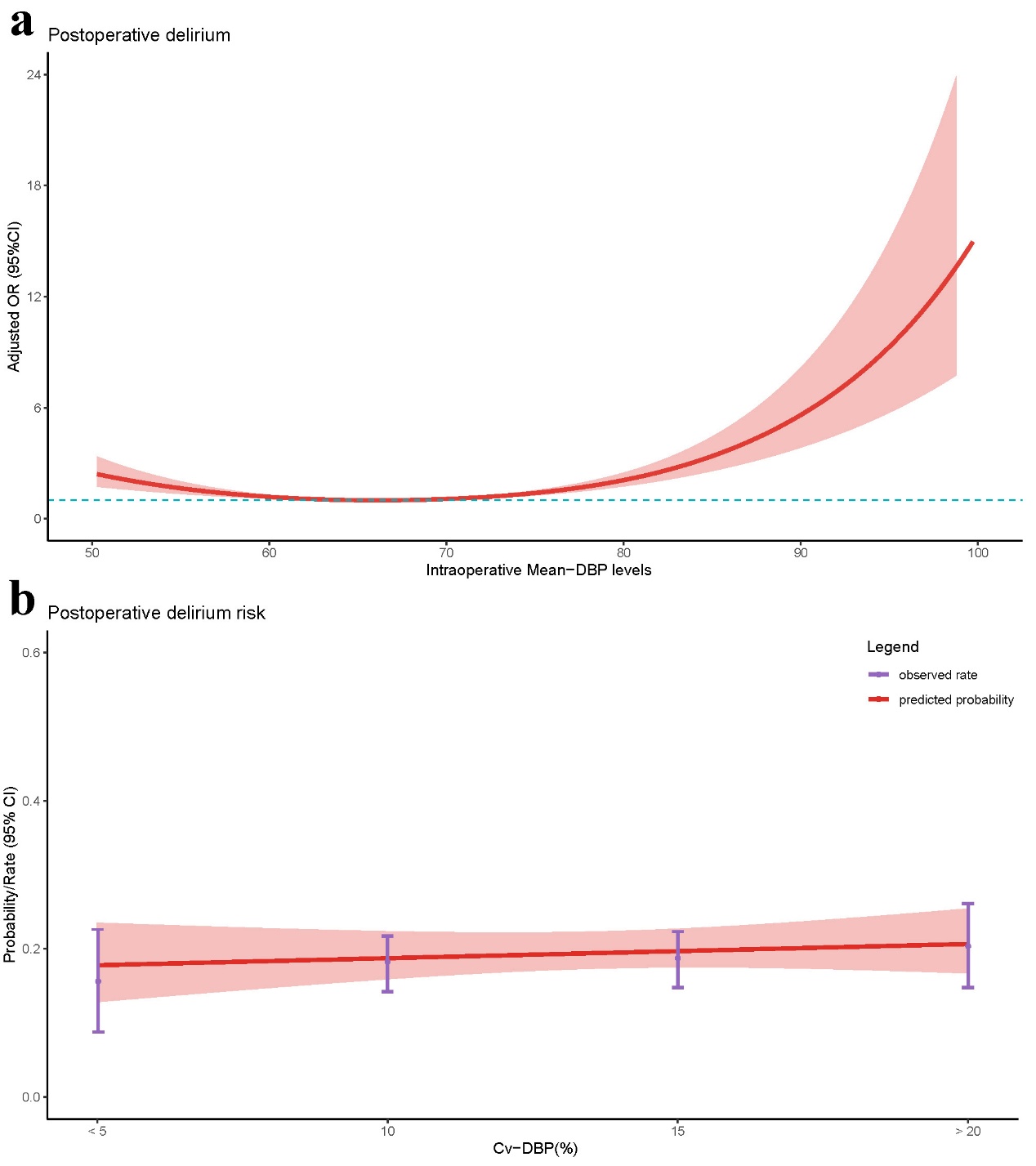


**eFigure 1** Relationship between intraoperative Mean− SBP levels and POD in patients with hip fracture. **(a)** Adjusted odds ratios (ORs) and 95% confidence intervals (CIs) are shown for 10 mmHg deviation away from the reference value of intraoperative Mean− SBP levels. **(b)** Predicted probabilities and the observed rate of POD based on CV-DBP.
